# Nanoscale control of phonon excitations in graphene

**DOI:** 10.1038/ncomms8528

**Published:** 2015-06-25

**Authors:** Hyo Won Kim, Wonhee Ko, JiYeon Ku, Insu Jeon, Donggyu Kim, Hyeokshin Kwon, Youngtek Oh, Seunghwa Ryu, Young Kuk, Sung Woo Hwang, Hwansoo Suh

**Affiliations:** 1 Device Lab., Samsung Advanced Institute of Technology, Suwon 443-803, Korea; 2 Department of Mechanical Engineering, Korea Advanced Institute of Science and Technology (KAIST), Daejeon 305-701, Korea; 3 Department of Physics and Astronomy, Seoul National University, Seoul 151-747, Korea

## Abstract

Phonons, which are collective excitations in a lattice of atoms or molecules, play a major role in determining various physical properties of condensed matter, such as thermal and electrical conductivities. In particular, phonons in graphene interact strongly with electrons; however, unlike in usual metals, these interactions between phonons and massless Dirac fermions appear to mirror the rather complicated physics of those between light and relativistic electrons. Therefore, a fundamental understanding of the underlying physics through systematic studies of phonon interactions and excitations in graphene is crucial for realising graphene-based devices. In this study, we demonstrate that the local phonon properties of graphene can be controlled at the nanoscale by tuning the interaction strength between graphene and an underlying Pt substrate. Using scanning probe methods, we determine that the reduced interaction due to embedded Ar atoms facilitates electron–phonon excitations, further influencing phonon-assisted inelastic electron tunnelling.

## Abstract (web summary)

A fundamental understanding of electron–phonon interactions in graphene is crucial to developing graphene-based electronic and photonic devices. Here, the authors control the properties of phonons in graphene by tuning the interaction strength between graphene and an underlying platinum substrate.

## Graphical abstract

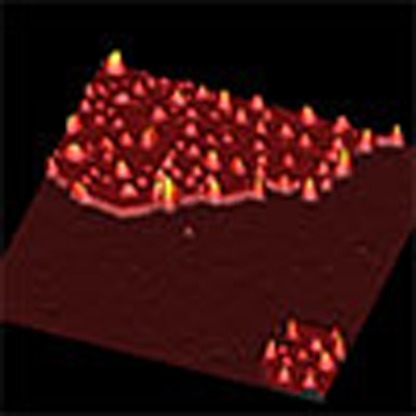

Graphene, which is known to exhibit linear energy dispersion near the Fermi level, is considered a highly desirable material for future electronic^1^,^2^,^3^ and photonic^4^,^5^ devices owing to its unique electronic properties. The peculiar electronic band structure of graphene is markedly modified by many-body interactions^6^ such as electron–electron, electron–plasmon and electron–phonon interactions^7^,^8^. Phonons in graphene can be excited by tunnelling electrons from a tip through the use of scanning tunnelling microscopy (STM), and the electron–phonon coupling is revealed as an enhancement of electron tunnelling to graphene states with energies larger than the phonon energy. And these modifications manifest as a gap-like feature at the Fermi level in the tunnelling spectra obtained via scanning tunnelling spectroscopy measurements^9^,^10^,^11^,^12^. If controllable, therefore, phonons in graphene may present a valuable opportunity and play an integral role in graphene-based device applications, for example, as a ‘floodgate'^9^ of electronic current. However, very few studies have systematically explored the generation and control of phonons in graphene, and thus far, it has only been speculated that the phonon excitations may be influenced by the interaction between graphene and the underlying substrate^10^,^11^,^12^.

In the following, we propose a simple method to fabricate deformed graphene for easy modification of the graphene–substrate interaction with local control. We choose nanobubbles as the structural ‘knob', that is, an on-off switch for phonon control, and exploit the fact that noble-gas ions can penetrate below the first atomic layer of a graphite surface to form interstitial defects by low-energy impinging^13^,^14^,^15^.

## Results

### Formation of graphene nanobubbles

**Figure 1:**
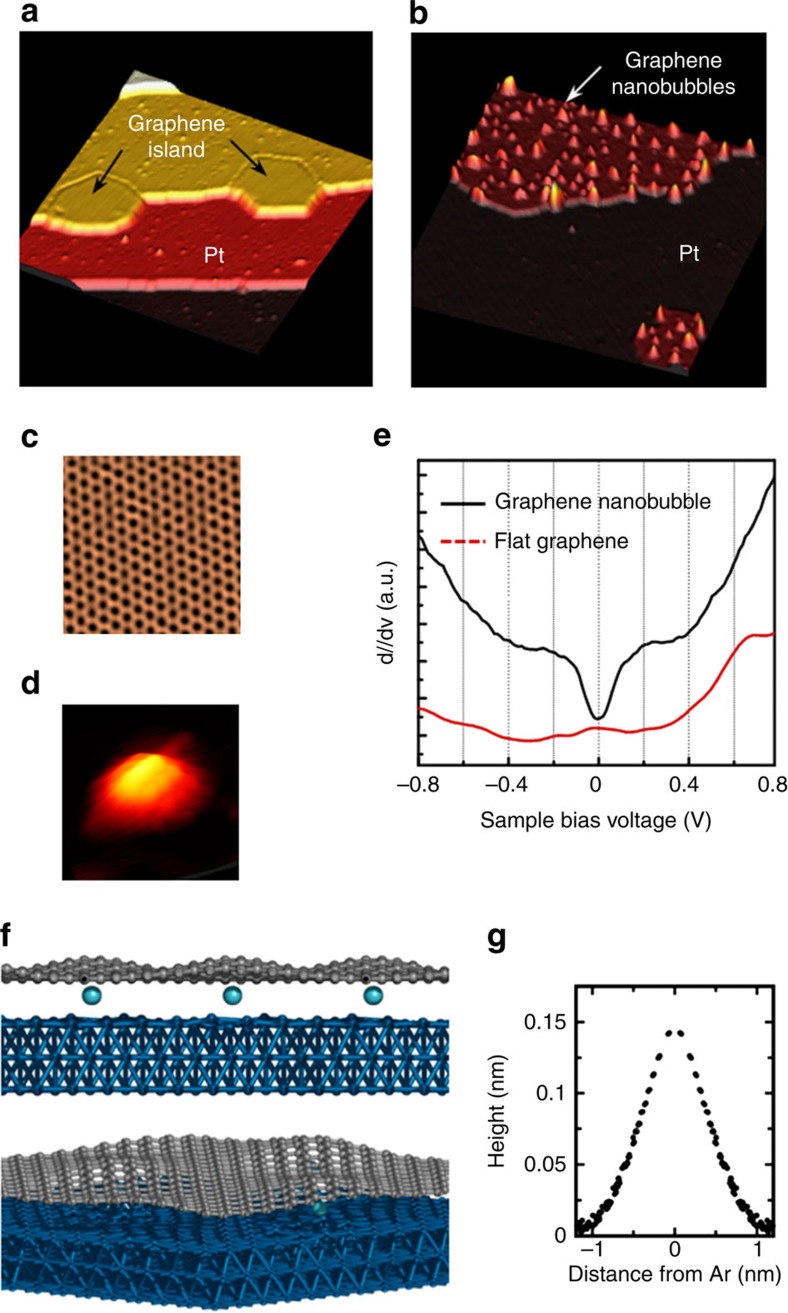
Graphene nanobubbles on Pt(111). (**a**) STM topograph of graphene nanoislands grown on Pt(111) (area of 50 × 50 nm^2^, *V*_s_=0.5 V, *I*_t_=0.5 nA). (**b**) STM topograph of graphene nanobubbles formed on graphene nanoislands (area of 25 × 25 nm^2^, *V*_s_=0.5 V, *I*_t_=1 nA). (**c**,**d**) Close-up STM topograph of the graphene honeycomb lattice (area of 2.8 × 2.8 nm^2^, *V*_s_=0.4 V, *I*_t_=30 nA) and nanobubble (area of 3.0 × 3.0 nm^2^, *V*_s_=0.03 V, *I*_t_=30 nA), respectively. (**e**) d*I/*d*V* spectra obtained at a graphene nanobubble (black line) and the flat graphene area (dotted red line). To make gap more evident, we added offset to the d*I/*d*V* spectrum measured in the flat graphene area. (**f**) Density functional theory (DFT)-optimized structure (side and tilted top views) of Ar-implanted graphene on Pt (111). The blue, grey and cyan spheres represent Pt, C and Ar atoms, respectively. (**g**) *z*-direction displacement of each C atom from a reference atom plotted against *x*–*y*-plane-projected distance from the closest Ar atom. The reference atom is selected to be the C atom closest to the Pt surface, and each point is duplicated for the corresponding negative distance.

In our study, graphene nanoislands were first grown on a Pt(111) substrate. The large-scale topography (Fig. 1a) as well as the atomic-scale honeycomb lattice of graphene (Fig. 1c) was clearly observed via STM. On controlled irradiation by Ar^+^ ions, protrusions emerged as a result of certain modifications in graphene but not in Pt(111), as evident in Fig. 1b. The atomistic corrugation in the close-up STM topography (Fig. 1d) confirms changes in the structural and electronic properties in the irradiated graphene. The experimentally observed protrusions in the Ar^+^-ion irradiated graphene can in general be attributed to several origins such as adatom adsorption, vacancy-induced deformation^16^ and nanobubble formation by noble-gas-atom implantation^13^,^14^,^15^. In this context, we carefully eliminated the possibilities of adatoms and carbon vacancies as the reasons for the observed protrusions by controlling the energy and angle of Ar^+^-ion sputtering with subsequent annealing. This is evidenced by the fact that the generally observed resonance peak near the Dirac point, which is characteristic of vacancies in graphene, is indeed absent in the differential conductance (d*I/*d*V*) spectrum^16^ (Fig. 1e). Instead, the presence of the aforementioned gap-like feature near the Fermi energy clearly indicates that nanobubbles are selectively generated using our method. Density functional theory (DFT) calculation results also confirmed the structural deformation and the formation of nanobubbles in graphene on Ar-atom implantation between graphene and the Pt surface. As shown in Fig. 1f, graphene ‘bends' around the intercalated atoms and forms structures resembling the experimentally observed nanobubbles. With a single intercalated Ar atom, each nanobubble has approximate diameter and height of 2 nm and 0.15 nm, respectively (Fig. 1g), and these dimensions agree well with those of the smallest nanobubbles observed in our experiment. Our calculations also indicated that nanobubbles of various sizes can form with multiple Ar-atom implantations (see Supplementary Fig. 1).

### Size dependence of nanobubbles in phonon excitations

**Figure 2:**
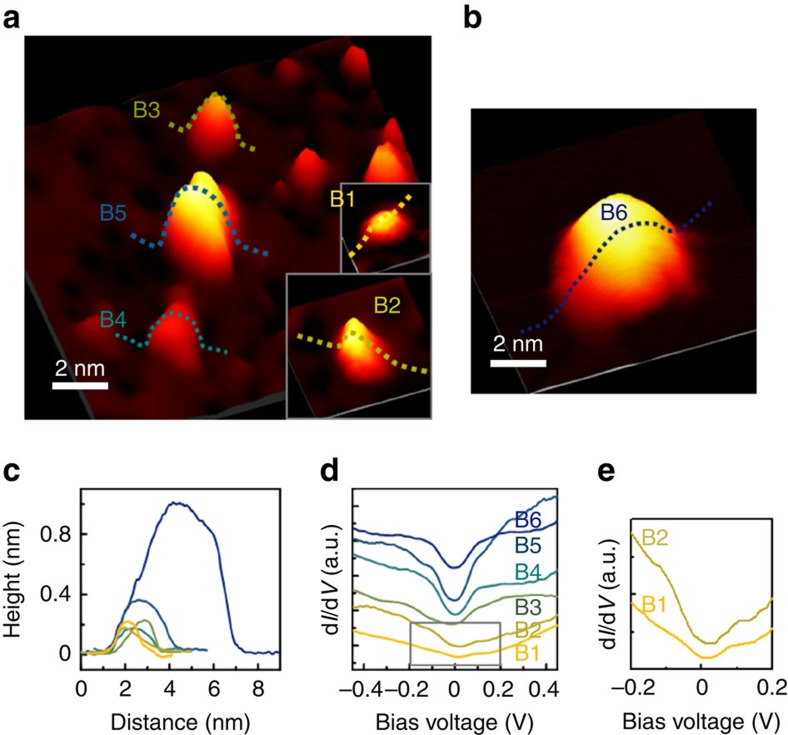
STM topograph of various graphene nanobubbles and their corresponding differential conductance (d*I/*d*V*) spectra. (**a**,**b**) STM topograph of graphene nanobubbles of various sizes. (*V*_s_=50 mV, *I*_t_=30 nA). (**c**) Dimensional cross-sections along the lines in **a**,**b**. (**d**) d*I/*d*V* spectra obtained at the centres of graphene bubbles B1–B6 shown in **a**,**b**. (**e**) Magnified d*I/*d*V* spectra near the Fermi level obtained at the centres of B1 and B2.

The bubbles labelled B1–B6 in Fig. 2a,b are representative examples of nanobubbles whose diameters and heights range from 1 to 6 nm and from 0.1 to 1.0 nm, respectively, as shown in the profiles (Fig. 2c) obtained along the dotted lines across B1–B6. The d*I/*d*V* spectra of the nanobubbles obtained near their apexes share the common gap-like feature centred at the Fermi level (Fig. 2d). While the ‘depth' of the gap and the slope of its edge increase monotonically with the size of the nanobubbles, the width and edge position of the gap exhibit no such dependence, which is further clearly evidenced by the magnified spectra of B1 and B2 in Fig. 2e.

The gap-like feature in the d*I/*d*V* spectra signifies the suppression and/or enhancement of electron tunnelling at specific bias voltages, which we conclude to originate from phonon excitations. In graphene, out-of-plane phonons at **K** points in Brillouin zone can generate an inelastic tunnelling channel, and the structural nature of the nanobubbles facilitates such phonon excitations and mediates inelastic tunnelling. As a result, a gap-like feature emerges near the Fermi energy^9^,^17^. Since the momentum and energy of the excited phonons are fixed, the gap size must be independent of the nanobubble size, which in fact agrees with our experimental results. Here we remark that similar derivative features in the d*I/*d*V* spectrum may also result from nanobubble-induced curvature. Structural strain modifies the electronic structure of graphene rather substantially, as evident in the example of the strain-induced pseudo-magnetic field manifesting as Landau level peaks in the d*I/*d*V* spectrum^11^,^18^,^19^,^20^. However, the strain-induced changes in the electronic structure depend on the amount of the strain, specifically on the size of nanobubbles in our case. The degree of shift in the position of the Landau level peaks has indeed been reported to vary depending on the curvature^11^,^19^, which contradicts our observation. Changes in the d*I/*d*V* spectrum may also arise from the embedded Ar atoms, which can locally dope the graphene to cause a shift in the Dirac point^21^,^22^ or generate additional states to appear as peaks or dips in the tunnelling spectra. Such changes again must depend on the number of Ar atoms, or the size of the nanobubbles, which disagrees with our experiment. Our DFT calculations indeed did not show any discernible changes in the local density of states subsequent to the formation of nanobubbles, thus indicating that no additional charge states were present (See Supplementary Fig. 2).

**Figure 3:**
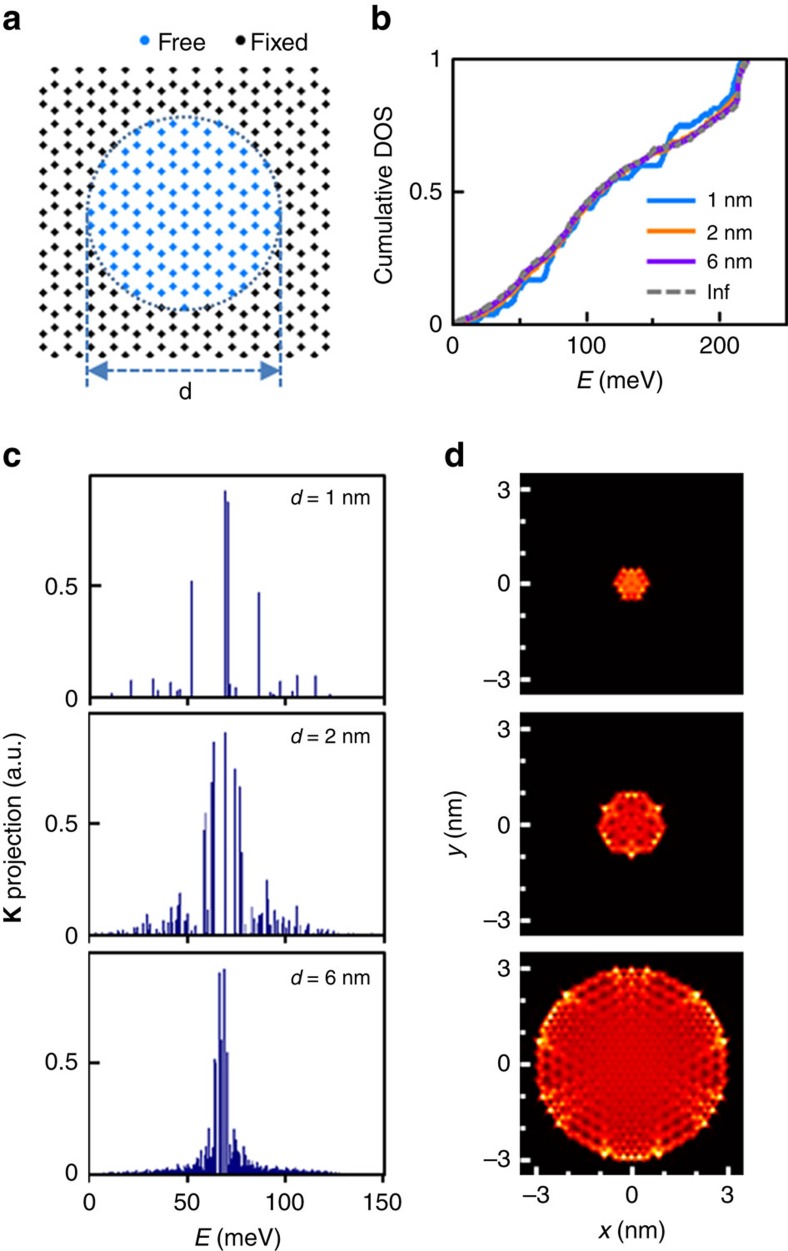
Calculation of phonon modes in nanometre-size graphene dots. (**a**) Schematic of graphene dot of diameter *d*, wherein the atoms inside the dot are free to move while those outside are stationary. (**b**) Graph of cumulative phonon density of states (DOS) for graphene dots with different diameters. All graphs are normalized so that the maximum cumulative phonon density of state (CDOS) is 1. (**c**) Projection of each phonon mode to a plane wave with momentum **K** and out-of-plane displacements for graphene dots with *d*=1, 2 and 6 nm. (**d**) Root mean square (r.m.s.) amplitudes of the phonon modes with **K** projections larger than one-third of the maximum **K** projection for the *d* values shown in **c**.

We examined how the lateral size of a graphene nanobubble influences phonon excitations via phonon-mode calculations by using an atomistic model. In our experiment, phonon excitations were localized in nanobubbles with diameters as small as 1 nm (B1, Fig. 2), so we constructed a simple model of a circular graphene dot (Fig. 3a), which mimics the case wherein the graphene is fully attached to the metal outside the dot while being completely free-standing inside the bubble. The complete phonon modes were computed for graphene dots with various diameters *d*, and the resulting normalized cumulative phonon density of states is shown in Fig. 3b. For all values of *d*, the cumulative phonon density of states exhibit nearly identical distributions although discrete jumps are observed in the case of *d*=1 nm because of the quantisation effect. Our results demonstrate that phonons are indeed excitable in nanobubbles with lateral sizes of ∼1 nm. The dependence of the phonon energy distribution on the bubble size was also explored by calculating the projection of each out-of-plane phonon mode to the plane wave with momentum **K** (**K** projection). As shown in Fig. 3c, the maximum value of **K** projection occurs at 68–70 meV for all finite-size dots, whose energy range corresponds well with the phonon energy of 67.3 meV in an infinite plane with momentum **K**. However, the larger the dot size is, the narrower the calculated distribution of the **K** projection becomes, and this behaviour explains why the depth and the edge slope of the gap-like feature in the d*I/*d*V* are observed to increase for larger bubbles. In addition, the calculation of the root mean square amplitude of the phonon modes (Fig. 3d) shows a uniform distribution for all nanodot sizes, which indicates that phonon signals are expected to be detected throughout the bubble. We note that the local variations in the amplitude result from the √3 × √3 pattern that is specific to the momentum **K** (see Supplementary Fig. 3).

### Spatial distribution of phonon excitations

**Figure 4:**
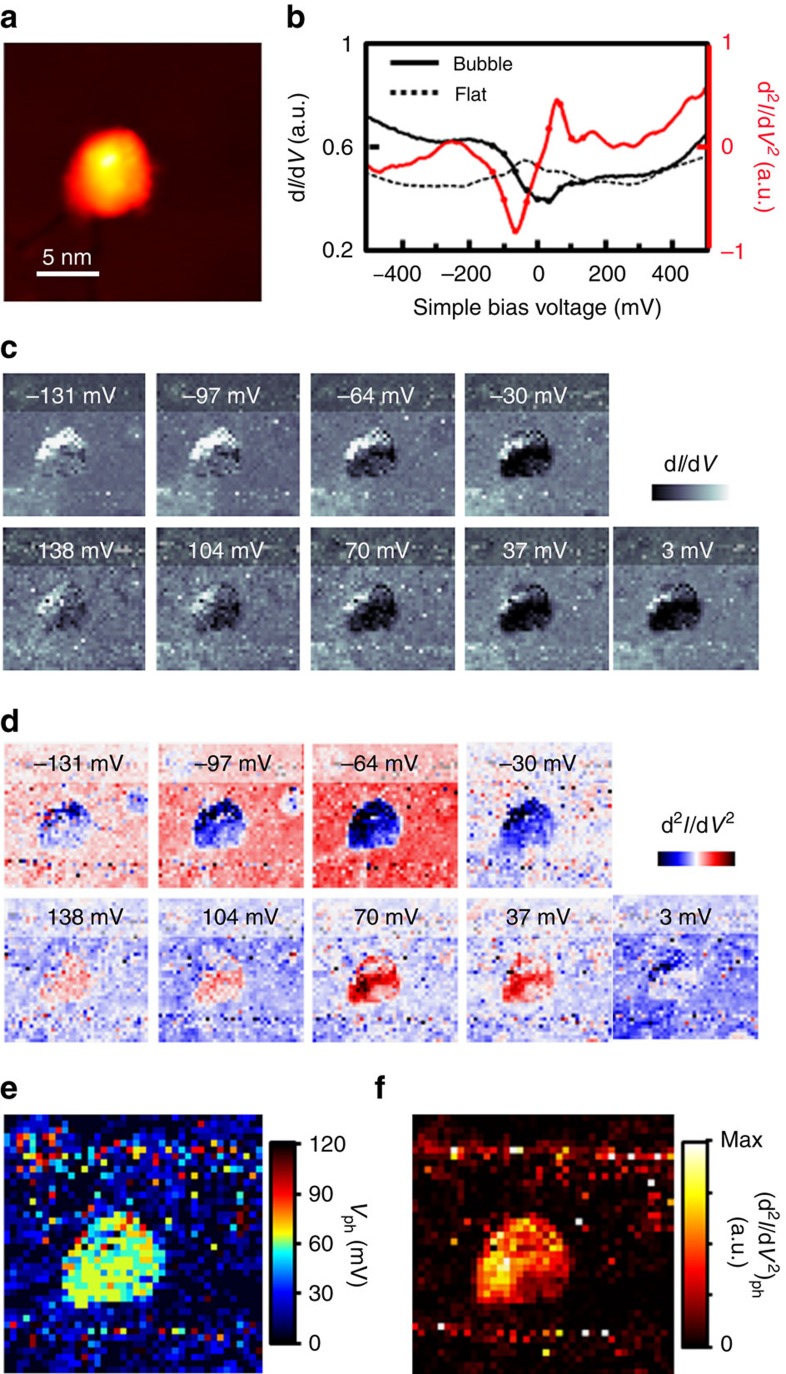
d*I/*d*V* and d^2^*I/*d*V*^2^ maps of nanobubble. (**a**) STM topograph of graphene nanobubble B6 (*V*_s_=500 mV, *I*_t_=1 nA). (**b**) Averaged d*I/*d*V* (black) and d^2^*I/*d*V*^2^ (red) spectra. The d^2^*I/*d*V*^2^ spectra were calculated by numerical derivatives of the corresponding d*I/*d*V* spectra. The solid line indicates values obtained within the nanobubble, while the dotted line indicates values obtained for the outer flat region. Along the solid line, the bias values at which the maps were plotted in **c**,**d** are marked by dots. (**c**) d*I/*d*V* and (**d**) d^2^*I/*d*V*^2^ maps obtained over the area shown in **a** for bias voltages *V*=−131, −97, −64, −30, 3, 37, 104 and 138 mV from left to right. (**e**) Map of the bias voltage of the largest d^2^*I/*d*V*^2^ peak, *V*_ph_, which corresponds to the local phonon energy. (**f**) Map of the amplitude of d^2^*I/*d*V*^2^ values at *V*_ph_, [d^2^*I/*d*V*^2^]_ph_, which corresponds to the local phonon intensity.

To examine the spatial distribution of excited phonons around graphene nanobubbles, we obtained the spatial d*I/*d*V* maps and the corresponding numerical derivative (d^*2*^*I/*d*V*^*2*^) maps. The spatial d*I/*d*V* map in Fig. 4c and the corresponding d^2^*I/*d*V*^2^ map in Fig. 4d around nanobubble B6 (Fig. 2b and Fig. 4a) clearly indicate that phonon excitation only occurs within nanobubbles which are detached from the graphene. The nanobubble region becomes ‘dark' for energies in the range −70 to +70 meV in the d*I/*d*V* maps because of the gap, and a strong contrast is observed at around ±70 meV in the d^2^*I/*d*V*^2^ maps, which is manifested as a peak and dip in d^2^*I/*d*V*^2^ spectra in Fig. 4b. The boundary of the phonon excitation in the d*I/*d*V* and d^2^*I/*d*V*^2^ maps coincides with that of the nanobubble in the topograph. We also plotted the bias voltages of the largest peak in the d^2^*I/*d*V*^2^ spectra, *V*_ph_, shown in Fig. 4e, and the *V*_ph_ shows little variation from 64 to 70 meV. However, the intensity of phonon signal increases as a function of the distance between the graphene and Pt layers, as plotted in Fig. 4f. The intensity increases by a factor of >2 when the separation between the Pt and graphene layers increases by 5 Å, which indicates that the phonon is more easily excited as the graphene–Pt interaction is reduced (see Supplementary Fig. 4). The exact mechanism by which the graphene–Pt interaction affects the phonon modes in nanobubbles are out of the scope of this paper; however, we note that the additional atomistic modelling demonstrates the phonon localization does occur by the change in the graphene–Pt interaction (see Supplementary Note 1, Supplementary Figs 5–7).

## Discussion

We successfully demonstrated phonon excitations in graphene via local control by generating nanobubbles of various sizes by means of Ar-ion irradiation. The reduced graphene–metal interactions due to Ar atoms embedded in between the graphene and metal substrate were shown to promote phonon excitations and enhance electron tunnelling above the phonon energy. We expect that our demonstrated control over phonon excitations down to the nanoscale will pave the way for advancements in graphene-based nano-electromechanical devices such as electrical switches based on the induced nonlinear *I–V* behaviour^9^. Our results also provide a fundamental understanding of the unwanted, yet possible, changes in electronic properties in actual graphene nanodevices, which arise from unwanted nanobubbles formed due to structural imperfections both in graphene and the supporting substrate.

## Methods

### Sample preparation and measurements

A Pt(111) sample was cleaned by means of repeated cycles of Ar-ion sputtering and annealing under ultrahigh vacuum. Graphene nanoislands were prepared by exposure of the clean Pt(111) surface to ethylene followed by annealing at 1,100 K^23^. To generate graphene nanobubbles, the graphene on the Pt(111) surface was irradiated with Ar^+^ ions (500 eV, 1 μA), and the sample was annealed at 1,000 K to remove additional impurities adsorbed onto graphene. Our STM measurements were performed using a Unisoku low-temperature STM at 2.8 K, and scanning tunnelling spectroscopy was performed using a conventional lock-in technique with a modulation bias voltage at a frequency of 1 kHz and an amplitude of 10 mV.

### Modelling

Our first-principles calculations are based on the DFT employing the generalized gradient approximation with the projector-augmented-wave method as implemented in the Vienna *ab initio* simulation package^24^,^25^,^26^, and the electronic wave functions are expanded in a plane wave basis set with a cut-off energy of 273.9 eV. The vdW-DF2 (ref. 27) functional is also selectively employed. The periodically replicated system of a 9 × 9 graphene supercell placed on a three-layer slab of 8 × 8 Pt(111) with a vacuum spacing of ∼10 Å, which reproduced the experimentally observed moiré pattern, was first optimized. A part of the carbon atoms in graphene was subsequently manually deformed to introduce an Ar atom in between the graphene and the metal surface. The atomic positions of the resulting structure were relaxed until residual forces were <0.02 eV Å^−1^.

### Atomistic calculation

We computed the Hessian matrix of the atoms within the graphene nanobubble using the adaptive intermolecular reactive empirical bond order (AIREBO) potential for carbon^28^, and we obtained the phonon spectra from the eigenvalues and eigenvectors of the Hessian matrix. We chose the AIREBO potential to avoid the otherwise large computational cost of first-principles calculations. While the phonon spectra from the AIREBO potential deviate slightly from the DFT calculations^29^, the AIREBO approach can correctly capture changes in the phonon spectra due to the boundary.

## Additional information

**How to cite this article:** Kim, H.W. *et al*. Nanoscale control of phonon excitations in graphene. *Nat. Commun*. 6:7528 doi: 10.1038/ncomms8528 (2015).

## Supplementary Material

Supplementary Information Supplementary Figures 1-7, Supplementary Note 1 and Supplementary References
