# Supplementary material for: Nanoscale control of phonon excitations in graphene
Source: Nat Commun. 2015 Jun 25;6:7528. doi: 10.1038/ncomms8528 (PMC4491832; doi:10.1038/ncomms8528)
Supplement: Supplementary Information — Supplementary Figures 1-7, Supplementary Note 1 and Supplementary References [file ncomms8528-s1.pdf]

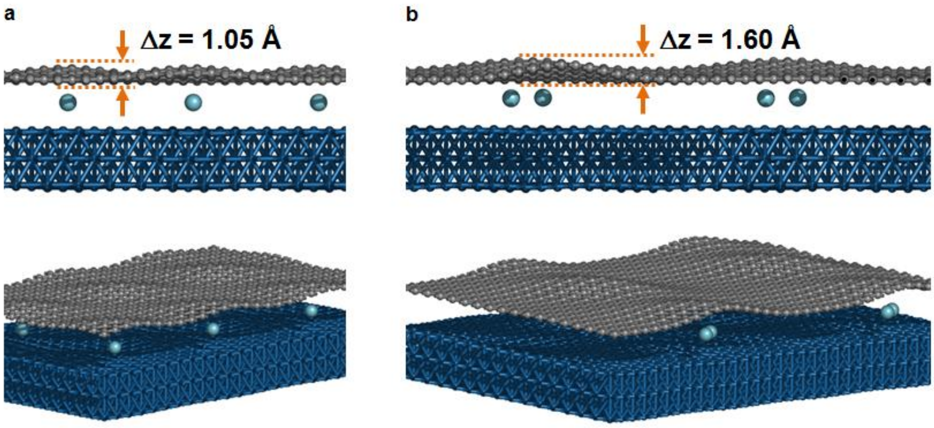

Supplementary Figure 1. Representative DFT-optimized structural models of Ar-embedded graphene on Pt(111). DFT calculation results employing GGA for model systems including (a) one or (b) two embedded Ar atom(s). As expected, graphene nanobubble size is clearly larger with more intercalated atoms. Exact nanobubble dimensions in (a) are different from those presented in Fig. 1f,g as vdW-DF2 functional use was omitted for calculation efficiency.

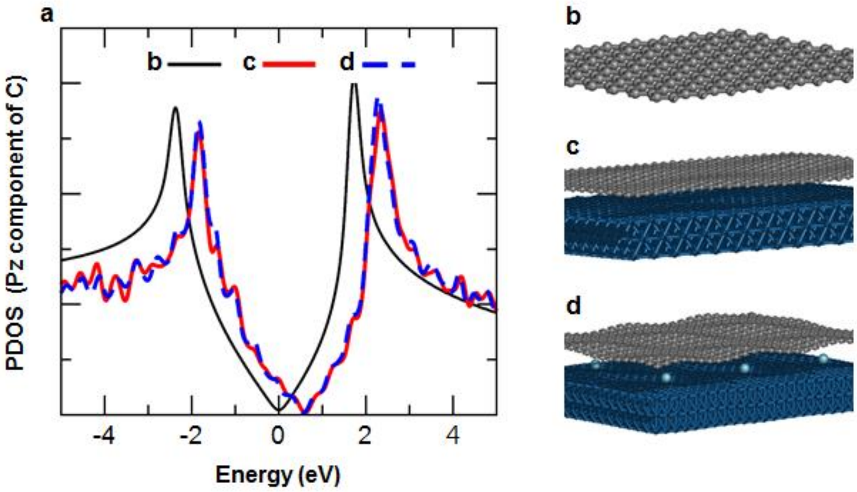

Supplementary Figure 2. (a) DFT-calculated projected density of states (Pz component of carbon) for (b) freestanding graphene, (c) graphene on Pt(111), and (d) graphene nanobubbles.

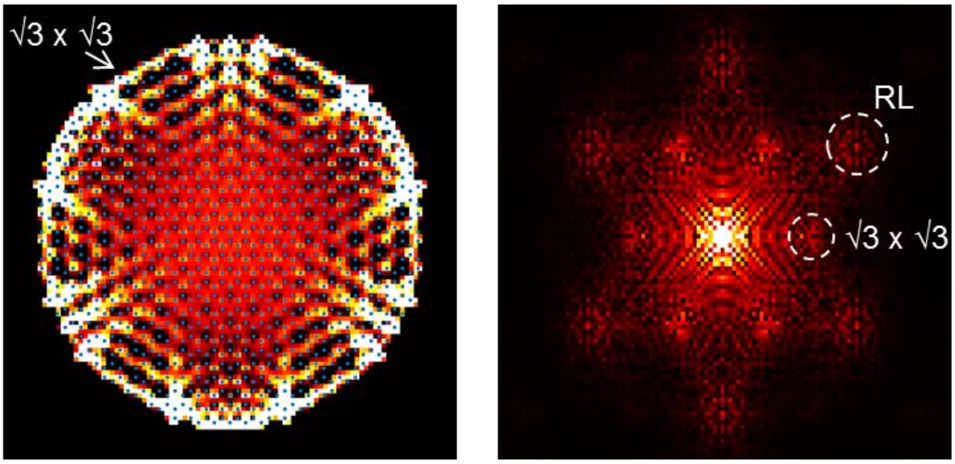

Supplementary Figure 3. Wavevector analysis of phonon modes. (Left) Phonon mode of d = 6 nm nanodot in Fig. 3d reproduced with higher contrast and with graphene lattice overlaid (cyan dots). Arrow indicates $\sqrt{3}\times\sqrt{3}$ pattern, which is strong at the edge. (Right) Fourier transform of the left image. Two sets of bright spots with six-fold symmetry are visible: one from the reciprocal lattice (RL) and the other from the $\sqrt{3}\times\sqrt{3}$ pattern.

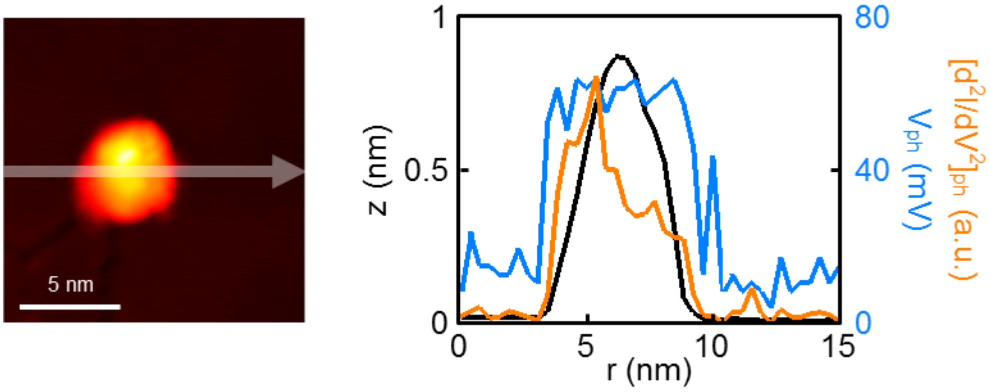

Supplementary Figure 4. Cross-section of nanobubble and corresponding phonon signal. (Left) Topograph of nanobubble (same as Fig. 4a). White arrow indicates location at which cross-section was taken. (Right) Cross-sections of height (black), phonon energy (cyan), and phonon intensity (orange) along nanobubble. Region in which phonon signal appears coincides with point at which nanobubble height starts to increase. Phonon energy is almost constant inside the nanobubble, while phonon intensity changes with height.

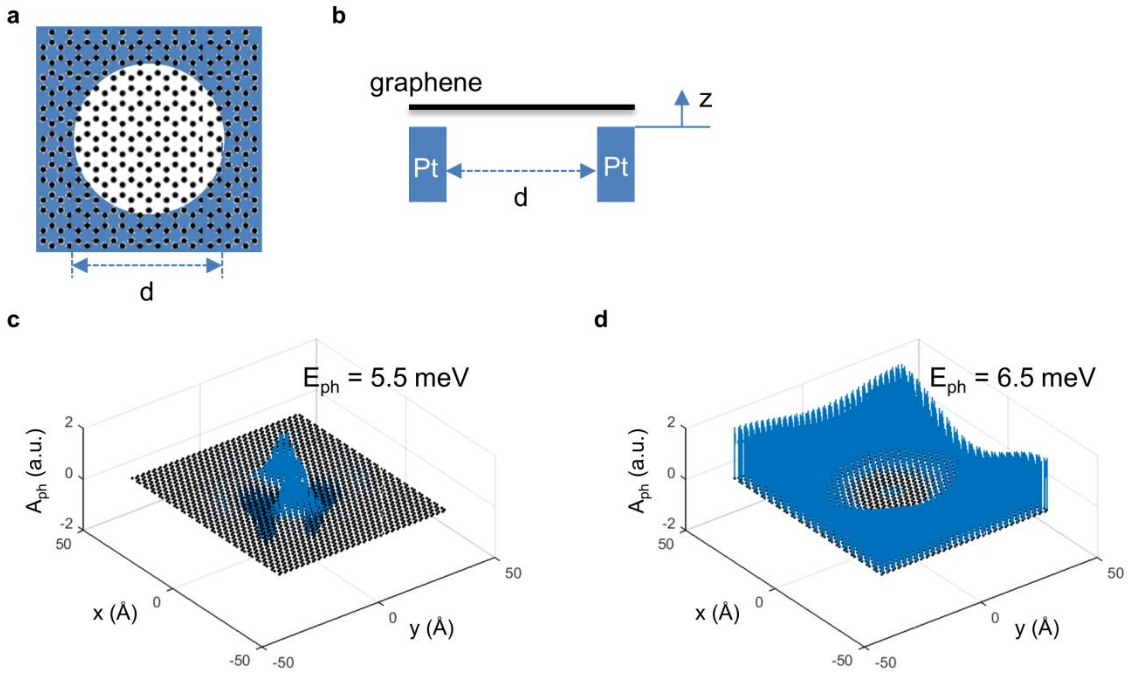

Supplementary Figure 5. Atomistic model of graphene dot including Pt substrate interaction. Schematics of graphene placed on Pt substrate with hole, viewed (a) from top and (b) from side. Black dots represent carbon atoms. Examples of phonon modes with $d = 2$ nm are plotted in (c) and (d), where modes are confined inside or outside of the hole, respectively. Phonon amplitudes are plotted as blue arrows on all carbon atoms.

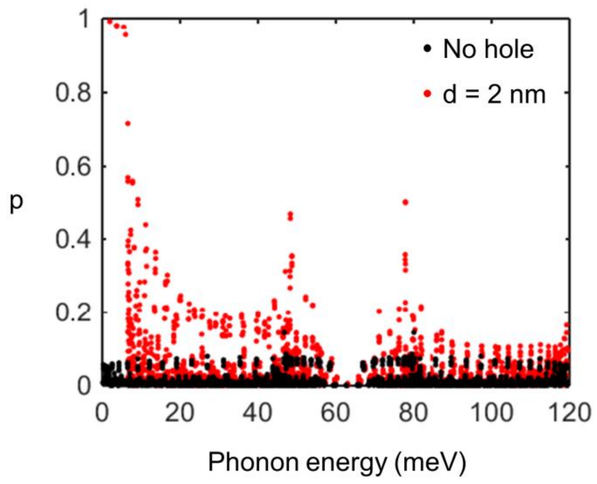

Supplementary Figure 6. Phonon localization effect. Degree of phonon localization effect *p* is plotted for all out-of-plane phonon modes, both for free-standing graphene (black) and for graphene on *d* = 2 nm hole (red).

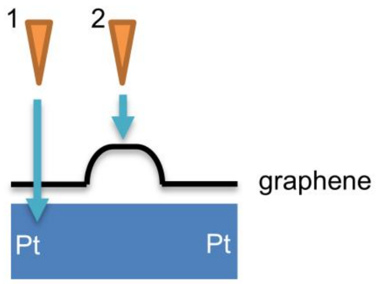

Supplementary Figure 7. Electron tunnelling process around the nanobubble. Schematic of electron tunnelling process for nanobubbles on Pt substrate. In case 1, electron tunnels mostly into Pt surface state. In case 2, electron tunnels mostly into graphene states.

Supplementary Note 1

Atomistic modelling of graphene phonon localization by graphene-substrate interaction

The graphene-Pt substrate interaction and its effects on phonons were investigated with atomistic modelling. We modelled the interaction between a carbon atom and Pt atom as a van der Waals interaction using the Lennar-Jones form, $E(r) = 4\epsilon[(\sigma/r)^{12}-(\sigma/r)^{6}]$, where $E(r)$ is the potential energy between the two atoms and ϵ and σ are the parameters determining the interaction strength and equilibrium distance, respectively. By integrating the potential energy of a Pt half-infinite surface, the total potential at the carbon atom by the Pt substrate can be written as $U(z) = \epsilon[2/15\ (\sigma/z)^{9}-(\sigma/z)^{3}]$, where z is the distance between the Pt substrate and the graphene layer. The values ϵ = 43.64 meV and σ = 3.705 Å are chosen to match the binding energy (46 meV/carbon atom) and equilibrium distance (3.18 Å) obtained from the first-principle calculation[1].

To investigate how the variation of $U(z)$ affects the phonons, we constructed a simple model in which the flat graphene was on top of the Pt substrate, which contained a hole with diameter d in the middle (Supplementary Figure 5a,b). For simplicity, we assumed that the carbon atoms on the Pt substrate were subject to the external potential $U(z)$, while the atoms within the hole remained free. To obtain the phonon modes from the model, we calculated the Hessian matrix from the finite displacement method. We first evaluated the forces on every atom in the graphene layer after displacing an atom by a small distance δ in one direction and then repeated the calculations for a negative displacement $-\delta$. Then, the force constants on each atom due to the displaced atom could be obtained from the numerical derivative using the central-difference formula with forces from positive and negative displacements. For N atoms and displacements in three (x, y, z) directions, we obtained a $3N\times3N$ Hessian matrix by

repeating the procedure $3N$ times. The normal modes and frequencies were obtained from the eigenvectors and eigenvalues of the Hessian matrix, respectively.

The difference between the potentials inside and outside of the hole induced a slight change of about 0.8% in the spring constant of the carbon-carbon bonds in the z direction. Although the magnitude of the change was tiny, we still observed the non-negligible localization effect on the phonon modes. The amplitude of the phonon modes becomes either more concentrated inside the hole or outside of the hole, when compared to the case of free standing graphene. The effect is most prominent for low energy phonon modes, and Supplementary Figure 5c,d show two most pronounced examples of the phonon modes in the $d = 2$ nm case. Our results indicate that even weak van der Waals interaction between the graphene and substrate can affect the phonon modes and localize them in the nanoscale region.

The degree of localization was different for different phonon modes. For quantitative analysis, we defined the degree of phonon localization effect p as

$$p = |A_{ph,in} - A_{ph,out}| \,/\, (A_{ph,in} + A_{ph,out}),$$

where $A_{ph,in}$ ($A_{ph,out}$) is the rms phonon amplitude inside (outside) the hole and p ranges from zero to one, where higher values denote increased localization. Supplementary Figure 6 shows p calculated for all phonon modes, both in free-standing graphene and graphene on a $d = 2$ nm hole. A drastic difference is evident for low-energy phonons; p is negligible for the phonon modes in free-standing graphene but is almost one for the modes in graphene on the $d = 2$ nm hole. The value of p decreases as phonon energy increases, but across the whole

energy range, *p* is higher for graphene on the hole than it is for free-standing graphene. In actual nanobubbles, the graphene is significantly warped and strained at the nanobubble edges, so the phonon localization effect would be even higher than that yielded by the model of Supplementary Figure 5.

Atomistic modelling shows that the change in the substrate interaction split the phonon modes into two categories: one confined inside the hole and the other outside of the hole. We only observed phonon modes confined inside the nanobubbles by STM. The reason for this observation is probably related to the tunnelling process of the electrons to the graphene on Pt(111). Recent studies have shown that graphene nanoislands on metal surfaces exhibit the surface states of the metal and not of the graphene, as is revealed by the confined states observed by STM[2]. The same phenomena were observed in a graphene nanoisland on a Pt(111) surface[3], so we suppose that the tunnelling electrons outside the bubble mostly tunnelled into the metal surface state and did not interact with the graphene phonons (case 1 in Supplementary Figure 7). However, when the graphene distance from the Pt increases due to Ar intercalation, tunnelling to the metal surface state should decrease exponentially, so electrons should begin to tunnel into the graphene states and excite the phonons (case 2 in Supplementary Figure 7).

Supplementary References

1. Gong, C., Lee, G., Shan, B., Vogel, E. M., Wallace, R. M., and Cho, K. First-principles study of metal-graphene interfaces. *J. App. Phys.* **108**, 123711 (2010).

2. Altenburg, S. J., Kröger, J., Wehling, T. O., Sachs, B., Lichtenstein, A. I., and Berndt, B., Local Gating of an Ir(111) Surface Resonance by Graphene Islands, *Phys. Rev. Lett.* **108**, 206805 (2012).

.
